# Parent–Child Conflict and Psychological Adjustment: The Serial Mediating Roles of Psychological Control and Basic Psychological Needs

**DOI:** 10.3390/bs16071055

**Published:** 2026-06-25

**Authors:** Mingshu Chen, Wan Ding, Jingning Liu, Ningxin Su

**Affiliations:** 1 Zhejiang Philosophy and Social Science Laboratory for the Mental Health and Crisis Intervention of Children and Adolescents, School of Psychology, Zhejiang Normal University, Jinhua 321004, China; 2 Joint Education Institute of Zhejiang Normal University and University of Kansas, Zhejiang Normal University, Jinhua 321004, China

**Keywords:** parent–child conflict frequency, parental psychological control, basic psychological needs, psychological adjustment

## Abstract

Although existing research has found that parent–child conflict significantly predicts children’s psychological adjustment, it remains unclear whether father–child and mother–child conflict exert distinct effects on psychological adjustment, the mediating processes through which they operate, and whether these processes vary across primary and secondary school stages. Using a three-wave longitudinal design, this study examined 1210 primary school students (*M*_age_ = 10.17, *SD*_age_ = 0.85) and 973 secondary school students (*M*_age_ = 12.62, *SD*_age_ = 1.36). A multiple mediation model integrating parallel and serial paths was constructed to investigate how father–child and mother–child conflict frequency respectively predicted four indicators of psychological adjustment (internalizing problems, externalizing problems, life satisfaction, and prosocial behavior) and to test the mediating roles of parental psychological control and basic psychological needs. Results showed the following: (1) parental psychological control and basic psychological needs served as significant independent mediators of the relationship between conflict frequency and psychological adjustment. In primary school, maternal psychological control emerged as the core mediator; in secondary school, the mediating role of paternal psychological control was significantly strengthened, and the basic psychological need mediated all associations between mother–child conflict and every adjustment indicator. (2) The serial mediating pathway “parental psychological control → basic psychological needs” was robust across both school stages. As a distal family stressor, parent–child conflict is indirectly transformed into maladjustment through a sequential process that first elevates psychological control and then thwarts basic psychological need. These findings illuminate a cascading mechanism underlying the impact of parent–child conflict on multifaceted adjustment and offer stage-specific guidance for targeted family interventions in primary and secondary school settings.

## 1. Introduction

Psychological adjustment in children and adolescents has long been a core topic in developmental and educational psychology. Psychological adjustment is typically defined as an individual’s active adaptation to internal and external demands, which covers multiple dimensions including emotions, behaviors, social functioning, and subjective well-being (Cicchetti & Rogosch, 2002). Among the multiple environmental systems that shape adjustment, the family is unquestionably the most critical socialization context. The frequency of parent–child conflict, a central risk indicator of family interaction quality (Barber et al., 2005), has been consistently linked to adjustment outcomes such as internalizing problems, externalizing problems, life satisfaction, and prosocial behavior (e.g., Moed et al., 2015; Zhu et al., 2020). However, most prior studies have treated parent–child conflict as a global construct, seldom differentiating the unique contributions of fathers and mothers, and have rarely clarified the mechanisms through which parent–child conflict contributes to children’s diverse adjustment outcomes.

According to family stress theory, family stressors do not influence psychological adjustment through a direct, singular pathway; instead, they operate indirectly by reshaping parenting behaviors and intraindividual psychological processes (Patterson, 2002; Xie et al., 2026). Thus, parent–child conflict frequency, as a family stressor, may indirectly affect adjustment via its impact on parenting. In parallel, self-determination theory holds that healthy development depends on the satisfaction of three basic psychological needs—autonomy, competence, and relatedness—and that environmental factors promote or undermine adjustment precisely by affecting the degree to which these needs are fulfilled (Deci & Ryan, 2000). Consequently, parent–child conflict frequency may also exert indirect effects on adjustment through basic psychological need. Within the family context, parental psychological control, a maladaptive parenting practice, is widely regarded as a key proximal factor that frustrates children’s basic need satisfaction (Soenens & Vansteenkiste, 2010). Synthesizing these perspectives, parent–child conflict may frequency initially evoke greater parental psychological control, which subsequently erodes basic psychological need, ultimately impairing their multidimensional adjustment through this serial pathway. However, to the best of our knowledge, no studies have yet examined whether parental psychological control and basic psychological needs mediate the relationship between parent–child conflict and children’s psychological adjustment, nor have any studies tested the existence of the chain mediating effect of “parent-child conflict → parental psychological control → basic psychological needs → psychological adjustment”.

Primary and secondary school students occupy distinct critical periods of development: primary school children’s emotions and behaviors remain heavily contingent on the immediate family climate, whereas secondary school students simultaneously face surging autonomy needs and the renegotiation of parent–child authority (Smetana et al., 2006; Zimmermann & Iwanski, 2014). This suggests that the mechanisms linking parent–child conflict to psychological adjustment may differ markedly across school stages, which remains to be examined.

Grounded in an integrated framework of family stress theory and self-determination theory, this study examined the longitudinal effects of father–child and mother–child conflict frequency on internalizing problems, externalizing problems, life satisfaction, and prosocial behavior among primary and secondary school students. It further tested the independent and serial mediating roles of parental psychological control and basic psychological needs.

### 1.1. Parent–Child Conflict Frequency and Psychological Adjustment

Parent–child conflict frequency is a key indicator of negative interaction dynamics, and extensive research has documented its robust associations with multidimensional psychological adjustment. With respect to internalizing problems, frequent parent–child conflict is a salient risk factor for children’s depression and anxiety (Hogye et al., 2022; Xie et al., 2024). For externalizing problems, Moed et al. (2015) demonstrated through observational data that the duration of parent–child conflict positively predicted secondary school students’ externalizing behaviors. The negative link between parent–child conflict and children’s life satisfaction has also received wide support (Zhu et al., 2020). In the domain of prosocial behavior, sustained conflict depletes children’s sensitivity to others’ needs, dampens empathic responding and willingness to help, and diverts psychological energy toward self-defense and relationship repair, thereby inhibiting the development of prosocial tendencies (Carlo et al., 2018). Taken together, prior evidence indicates that parent–child conflict frequency significantly predicts internalizing and externalizing problems, life satisfaction, and prosocial behavior.

Importantly, father–child and mother–child conflict may differ in both the magnitude and mechanism. Fathers and mothers assume distinct roles, interaction styles, and emotional expressions in family life, and children hold asymmetric expectations and perceptions of these two relationships (Lamb & Lewis, 2013). Accordingly, the present study differentiated parent–child conflict frequency into father–child and mother–child dimensions to examine their respective predictive effects on the four adjustment indicators.

### 1.2. The Mediating Role of Parental Psychological Control

Parental psychological control may mediate the association between parent–child conflict frequency and children’s psychological adjustment. Psychological control is a parenting practice in which parents manipulate children’s thoughts, emotions, and behaviors through pressuring, love withdrawal, and guilt induction, stifling their autonomy and enforcing compliance (Q. Wang et al., 2007). First, parent–child conflict frequency may positively predict parental psychological control. According to family systems theory, family subsystems are mutually influential, and tension in the parent–child subsystem can spill over into parenting behaviors (Cox & Paley, 1997). In other words, as a core marker of parent-child subsystem disequilibrium, conflict not only directly impairs interaction quality but may also alter parenting orientations. Frequent conflict can undermine parents’ sense of competence (“I cannot effectively discipline my child”) and relatedness needs, leading them to adopt controlling strategies such as guilt induction and love withdrawal to regain a sense of control over the parenting situation (Mabbe et al., 2018; Soenens & Vansteenkiste, 2010). Simultaneously, high conflict erodes parent–child trust and communication quality, making autonomy-supportive approaches more difficult to sustain and pushing interaction patterns toward a control orientation (Grolnick, 2003; L. Sun et al., 2021). A cross-cultural longitudinal study by Lansford et al. (2014) directly examined this link and found that the quality of negative parent–child interactions, including the conflict frequency, significantly predicted subsequent parental psychological control.

Second, once established, parental psychological control broadly impairs children’s adjustment. Drawing on self-determination theory, psychological control thwarts children’s need for autonomy, rendering authentic self-expression difficult (Deci & Ryan, 2000). At the emotional and behavioral level, it may exacerbate internalizing problems by intensifying self-criticism and rumination or provoke anger and defiance by obstructing autonomy, thus fostering externalizing problems (Soenens & Vansteenkiste, 2010; Wu et al., 2022). Zhou et al. (2025) further demonstrated a significant positive association between parental psychological control and internalizing problems among secondary school students, with chronically high-exposure students showing markedly elevated internalizing problems. At the social functioning level, psychological control suppresses children’s autonomous exploration and authentic emotional connections, diminishing positive attitudes toward others and willingness to help, thereby reducing life satisfaction and prosocial tendencies (Mabbe et al., 2018). Zhang et al. (2022) found that parental psychological control significantly and negatively predicted prosocial behavior in primary school children.

Taken together, existing research indicates that parental psychological control significantly predicts children’s adjustment outcomes, including internalizing problems, externalizing problems, life satisfaction, and prosocial behavior. Grounded in evidence that parent–child conflict frequency predicts parental psychological control and that psychological control predicts adjustment, the present study hypothesized that parental psychological control would significantly mediate the effect of parent–child conflict frequency on psychological adjustment.

### 1.3. The Mediating Role of Basic Psychological Needs

Self-system theory posits that basic psychological needs serve as the core mechanism linking the external environment to self-development (Connell & Wellborn, 1991). Self-determination theory further specifies that autonomy, competence, and relatedness are three indispensable basic psychological needs for healthy growth (Deci & Ryan, 2000). Thus, beyond the interpersonal pathway of parenting behaviors, parent–child conflict frequency may also indirectly affect psychological adjustment through the intrapersonal pathway of children’s basic psychological needs.

First, frequent parent–child conflict, as a prototypical relational stressor, may thwart the satisfaction of these needs in multiple ways. In conflict situations, children’s feelings and wishes are often ignored or negated, frustrating their need for autonomy; frequent criticism and blame convey negative competence feedback, undermining their sense of competence; ongoing conflict erodes parent–child emotional bonds, damaging their sense of relatedness (La Guardia & Patrick, 2008). Second, basic psychological needs are a key proximal predictor of psychological adjustment. When needs are thwarted, individuals may either shift toward compensatory goals such as external validation or lose positive motivational energy, thereby precipitating adjustment problems. Specifically, autonomy frustration is closely linked to internalizing problems such as depression and anxiety; competence frustration often manifests as both helplessness (internalizing) and impulsive aggression (externalizing); relatedness frustration reduces positive expectations for interpersonal interactions, diminishing prosocial behavior and lowering life satisfaction (Vansteenkiste & Ryan, 2013). Thus, parent–child conflict is likely to influence children’s psychological adjustment through the satisfaction of basic psychological needs.

Prior research with Chinese primary and secondary school students has verified the mediating role of basic psychological need in the influence of environmental factors on students’ adjustment. J. Hu and Zhou (2025) found that basic need satisfaction fully mediated the association between environmental stress and depression among secondary school students, with greater need frustration predicting higher depression. For prosocial behavior, Li et al. (2019) demonstrated that basic need satisfaction positively predicted prosocial behavior in secondary schoolers and mediated the link between family functioning and prosocial behavior. Regarding life satisfaction, P. Sun et al. (2022) found that basic need satisfaction was a proximal predictor of life satisfaction and mediated the effects of environmental variables such as peer relationships.

Taken together, the present study hypothesized that parent–child conflict frequency would exacerbate children’s internalizing and externalizing problems and diminish their life satisfaction and prosocial behavior by reducing basic psychological need satisfaction.

### 1.4. The Serial Mediating Effect of Parental Psychological Control and Basic Psychological Needs

In the pathway from parent–child conflict frequency to psychological adjustment, the interpersonal mediating process through parental psychological control and the intrapersonal mediating process through basic psychological needs may not operate independently; rather, they likely form a sequential serial mediation. According to self-determination theory (Deci & Ryan, 2000; Soenens & Vansteenkiste, 2010), psychological control impairs adjustment largely by undermining basic psychological need. Frequent parent-child conflict creates a chronic family stress context that increases parents’ use of psychological control strategies, which in turn erode students’ experience of autonomy (individual choices and volitions negated), competence (receiving messages of incapability), and relatedness (Deci & Ryan, 2000; Barber et al., 2012). Thwarted basic psychological needs then serve as a core proximal factor for adjustment problems such as internalizing difficulties, externalizing problems, and diminished life satisfaction (Costa et al., 2019; Vansteenkiste & Ryan, 2013; Wu et al., 2022). Stated differently, in families characterized by frequent parent–child conflict, psychological control represents a critical family interaction mechanism that frustrates basic psychological needs, creating a cascading chain from contextual stress to parenting behavior to internal need states that ultimately shapes psychological adjustment in primary and secondary school students. Accordingly, this study systematically examined the serial mediating role of parental psychological control and basic psychological needs in the relationship between parent–child conflict frequency and the four psychological adjustment indicators.

### 1.5. School Stage Differences

Primary and secondary school represent two qualitatively distinct periods in the developmental continuum, and examining the mechanisms of parent–child conflict separately by stage carries considerable theoretical and practical significance. During the primary years, children’s emotion regulation and social behavior remain highly dependent on the immediate secure base and attachment support provided by the family, rendering their adjustment indicators more proximally sensitive to fluctuations in the family environment (Cummings & Davies, 2010; L. Wang et al., 2023). Upon entering secondary school, adolescents’ need for autonomy surges dramatically, and the pressure to transform the parent–child relationship from unilateral authority to bilateral negotiation reaches its peak (Guyer et al., 2016; Smetana et al., 2006). More critically, parental behavior patterns adjust dynamically with children’s age and school stage (Bardach et al., 2023); fathers, who function as relatively peripheral authority figures during childhood, gradually become key agents directly shaping adolescent socialization, and their parenting effects are markedly amplified in adolescence (Jhang, 2025; Levitt et al., 2020). It is therefore necessary to embed both school stages within a unified comparative framework, which not only helps reveal developmental shifts in the mechanisms through which parent–child conflict operates, but also provides a theoretical basis for stage-tailored, precision interventions in family education.

### 1.6. The Present Study

Grounded in family stress theory, parent–child conflict frequency, a core indicator of negative interaction processes, may adversely affect four key adjustment outcomes: internalizing problems, externalizing problems, life satisfaction, and prosocial behavior. Yet the mechanisms through which conflict frequency contributes to multidimensional adjustment remain insufficiently understood, particularly regarding the distinction between paternal and maternal roles within the Chinese cultural context. Integrating family stress theory and self-determination theory, this study constructed and tested a serial mediation model encompassing both interpersonal and intrapersonal pathways. The following hypotheses were examined: **H1**: Higher parent-child conflict frequency will be indirectly associated with poorer adjustment across all four indicators through greater parental psychological control. That is, parental psychological control will mediate the relationship between parent–child conflict frequency and adjustment outcomes. **H2**: Higher parent-child conflict frequency will be indirectly associated with poorer adjustment across all four indicators through lower basic psychological need satisfaction. That is, basic psychological need satisfaction will mediate the relationship between parent–child conflict frequency and adjustment outcomes. **H3**: Higher conflict frequency will be linked to greater psychological control, greater psychological control will predict lower need satisfaction, and lower need satisfaction will subsequently predict poorer adjustment. Thus, psychological control and need satisfaction will serially mediate the relationship between conflict frequency and adjustment. **H4**: The serial mediation pathways will differ significantly between primary and secondary school students.

To exclude confounding influences, students’ gender, age, and family socioeconomic status were controlled as covariates (Chapleski & Gresham, 2023; Steinberg, 2014; Conger & Conger, 2008). This study not only theoretically integrates family stress theory and self-determination theory, advancing an understanding of the hierarchical cascade from family interaction to parenting behavior to internal need states to adjustment outcomes, but also provides a scientific basis for designing targeted family education guidance.

## 2. Method

### 2.1. Participants

Using a three-wave longitudinal design with one-year intervals, this study recruited 1210 primary school families and 973 secondary school families through cluster sampling in southwestern and southeastern China. Due to constraints in the study launch timing, T1 data were actually collected at T2 via retrospective reports, asking participants to assess their typical state based on accurate recall within the previous year; T2 and T3 data were collected in real time. At T1, the primary sample had a mean age of 10.17 (*SD* = 0.85), with 53.37% boys and 34.1% only children; the secondary school sample had a mean age of 12.62 (*SD* = 1.36), with 46.8% boys and 30.7% only children. The study was approved by the Ethics Committee of Zhejiang Normal University (Approval No. ZSRT2023100, 9 October 2023). Written informed consent was obtained from students and parents prior to data collection, and all data were kept strictly confidential. Although T1 relied on retrospective reports, the limited recall window and the use of a clearly defined reference period enabled this approach to provide a reasonable approximation of a stable baseline for concrete, high-frequency, observable behaviors such as parent–child conflict (Hardt & Rutter, 2004). Similar retrospective designs continue to be employed in recent longitudinal and cohort studies (e.g., Görges et al., 2020; Pettitt et al., 2024), although we fully acknowledge this limitation and have addressed it explicitly in the limitations section.

### 2.2. Measures

#### 2.2.1. Frequency of Parent–Child Conflicts

Parent–child conflict frequency was measured using the Parent–Child Conflict Questionnaire (Fang & Dong, 1998). The 8-item scale covers conflict domains including academics, household chores, peer interactions, allowance use, daily routines, attire and hairstyle, family relationships, and personal privacy. Parents rated the frequency of conflicts with their child in each domain over the past six months on a 5-point scale (1 = never, 5 = several times a day). Higher total scores indicate greater conflict frequency. In this study, Cronbach’s *α* was 0.94 for parents of primary school students and 0.94 for parents of secondary school students.

#### 2.2.2. Parental Psychological Control

Parental psychological control was measured using the child-report version of the Parental Psychological Control Questionnaire, originally developed by Shek (2007) and revised by Nie (2018). The 10-item scale includes separate father (5 items) and mother (5 items) forms, covering dimensions such as guilt induction, love withdrawal, and authoritarian assertion. All items are positively worded and rated on a 5-point scale (1 = completely disagree, 5 = completely agree), with higher scores reflecting greater perceived parental psychological control. Cronbach’s *α* was 0.96 for primary school students and 0.97 for secondary school students.

#### 2.2.3. Basic Psychological Needs

Basic psychological needs were assessed using the Basic Psychological Needs Scale (Martela & Ryan, 2016). The 9-item scale comprises three dimensions: autonomy, competence, and relatedness, each with three items. Items are rated on a 4-point scale (0 = not at all true, 3 = somewhat true), with Items 4, 6, and 9 reverse-scored. Higher total scores indicate greater need satisfaction. In this study, Cronbach’s *α* was 0.80 for primary school students and 0.78 for secondary school students.

#### 2.2.4. Psychological Adjustment

Internalizing and externalizing problems and prosocial behavior were assessed with the Strengths and Difficulties Questionnaire (SDQ; Goodman, 1997). The 25-item scale covers five dimensions: emotional symptoms, conduct problems, hyperactivity, peer problems, and prosocial behavior. Internalizing problems were formed by summing emotional symptoms and peer problems and externalizing problems by summing conduct problems and hyperactivity. Items are rated on a 3-point scale (1 = not true, 3 = certainly true), with Items 7, 11, 14, 21, and 25 reverse-scored. Higher internalizing and externalizing scores indicate more problem behaviors, whereas higher prosocial scores indicate more positive behaviors. In the present study, Cronbach’s *α* for the SDQ was 0.88 (primary) and 0.82 (secondary school) by sample.

Life satisfaction was measured with the Satisfaction With Life Scale (SWLS; Diener et al., 1985). The 5-item scale uses a 7-point scale (1 = strongly disagree, 7 = strongly agree), with higher total scores reflecting greater life satisfaction. Cronbach’s *α* was 0.93 for primary school students and 0.92 for secondary school students.

### 2.3. Data Analysis

Data preprocessing, descriptive statistics, correlations, and regression analyses were performed using SPSS 25.0. Structural equation models were constructed in Mplus 8.0, and the serial mediation model was tested using the bootstrap method with 5000 resamples. If the 95% confidence interval (CI) for unstandardized indirect effects does not include zero, it indicates that the indirect effect is significant.

## 3. Results

### 3.1. Common Method Bias

To assess the potential influence of common method bias, an unmeasured latent method factor (ULMF) was added to a multi-trait model comprising all self-report items (Podsakoff et al., 2003; Rodriguez et al., 2016). Items were loaded on their respective trait factors (father–child conflict, mother–child conflict, paternal psychological control, maternal psychological control, basic psychological need, internalizing problems, externalizing problems, prosocial behavior, and life satisfaction) and simultaneously on an orthogonal common method factor. The bifactor model demonstrated acceptable fit, CFI = 0.93, TLI = 0.93, RMSEA = 0.036, 90% CI [0.035, 0.037], SRMR = 0.030. Standardized loadings on the method factor were consistently low (range = 0.03~0.19, *M* = 0.09) and mostly non-significant. The explained common variance (ECV) attributable to the nine trait factors was 0.91, indicating that over 90% of the common variance was substantive. These results suggest that common method bias does not pose a serious threat to the validity of the findings.

### 3.2. Descriptive Statistics and Correlations

Descriptive statistics and correlations for all variables are displayed in Table 1. Independent-samples *t* tests showed that, except for mother–child conflict frequency, paternal psychological control, and maternal psychological control (which did not differ significantly), primary and secondary school students differed significantly on all other variables (*t*s = −6.64 to 9.41, *p*s = 0.000 to 0.721). Correlation analyses indicated that T1 parent–child conflict frequency, T2 parental psychological control, T2 basic psychological needs, and all T3 psychological adjustment outcomes were significantly correlated with one another.

### 3.3. Measurement Invariance Testing

Prior to comparing structural paths across school stages, multi-group confirmatory factor analyses were conducted to test measurement invariance for all core instruments (see Table 2). For each scale—father–child conflict, mother–child conflict, paternal psychological control, maternal psychological control, basic psychological needs, internalizing problems, externalizing problems, prosocial behavior, and life satisfaction—configural, metric, and scalar invariance models were sequentially tested using established criteria (ΔCFI ≤ 0.01, ΔRMSEA ≤ 0.015; Chen, 2007; Cheung & Rensvold, 2002) across primary and secondary school groups. For all scales, the fit of the configural model was acceptable, and imposing equality constraints on factor loadings (metric invariance) and item intercepts (scalar invariance) did not result in a meaningful deterioration in fit. Specifically, across all instruments, ΔCFI remained below 0.01, ΔRMSEA did not exceed 0.015, supporting full scalar invariance. These results establish that the measurement properties of all scales are comparable across primary and secondary school students, justifying subsequent cross-group comparisons of structural parameters.

### 3.4. Model Analyses

**Figure 1:**
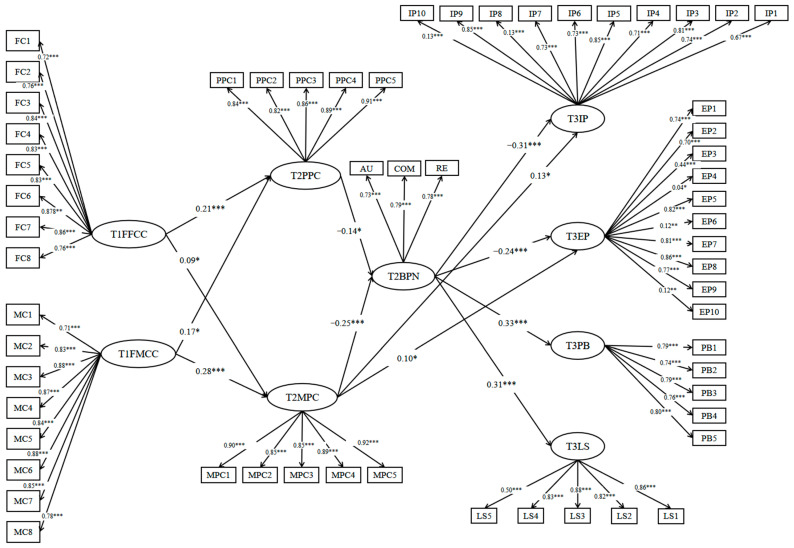
Significant model diagram of T1 parent–child conflict frequency predicting primary school students’ T3 psychological adjustment. Note: for model simplicity, only significant path coefficients are indicated, and control variables are omitted. FFCC = frequency of father–child conflicts, FMCC = frequency of mother–child conflicts, PPC = paternal psychological control, MPC = maternal psychological control, BPN = basic psychological needs, AU = autonomy need, COM = competence need, RE = relatedness need, IP = internalizing problems, EP = externalizing problems, PB = prosocial behavior, LS = life satisfaction. *** *p* < 0.001, ** *p* < 0.01, * *p* < 0.05.

**Figure 2:**
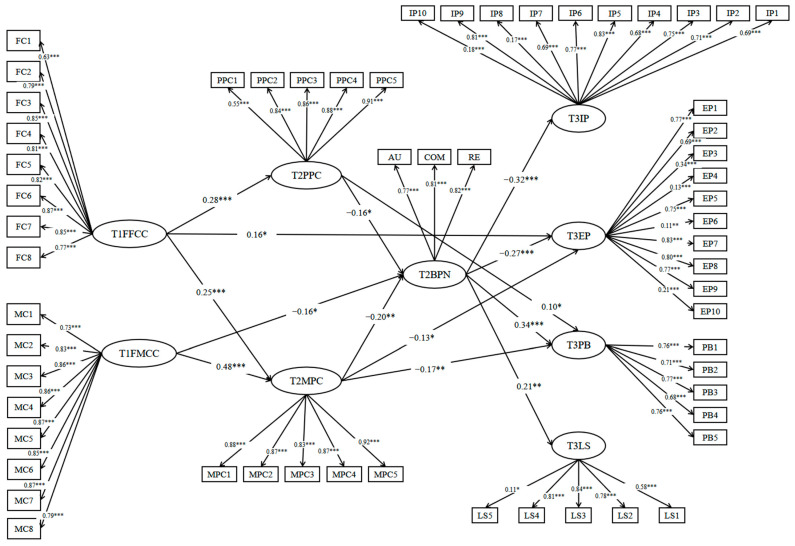
Significant model diagram of T1 parent–child conflict frequency predicting secondary school students’ T3 psychological adjustment. Note: for model simplicity, only significant path coefficients are indicated, and control variables are omitted. FFCC = frequency of father–child conflicts, FMCC = frequency of mother–child conflicts, PPC = paternal psychological control, MPC = maternal psychological control, BPN = basic psychological needs, AU = autonomy need, COM = competence need, RE = relatedness need, IP = internalizing problems, EP = externalizing problems, PB = prosocial behavior, LS = life satisfaction. *** *p* < 0.001, ** *p* < 0.01, * *p* < 0.05.

To address the potential impact of measurement error, a fully latent structural equation model was also estimated for each school stage (see Figure 1 and Figure 2). All constructs were specified as latent variables with their original items as indicators, except for basic psychological needs, which was represented by its three theoretically derived subscale scores (autonomy, competence, relatedness; Gagné, 2003). Additionally, three control variables (child gender, child age, family socioeconomic status) were included as covariates. For primary school students, the model demonstrated good fit: CFI = 0.94, TLI = 0.93, RMSEA = 0.04, SRMR = 0.06, χ^2^/*df* = 3.09. For secondary school students, the model demonstrated acceptable fit: CFI = 0.91, TLI = 0.91, RMSEA = 0.05, SRMR = 0.06, χ^2^/*df* = 3.21. Although the CFI for the secondary school model was slightly below the ideal threshold of 0.95, it remained above the commonly accepted cutoff of 0.90 (L. T. Hu & Bentler, 1999), and given the high model complexity (9 latent factors, 59 indicators, and 3 observed covariates), this level of fit was considered satisfactory (Kline, 2016).

For primary school students, T1 conflict frequency did not significantly predict any T3 adjustment outcomes directly. As shown in Table 3, for specific indirect effects, maternal psychological control significantly mediated the links between mother–child conflict frequency and internalizing problems (*β* = 0.04, 95% CI [0.010, 0.088]) and externalizing problems (*β* = 0.03, 95% CI [0.007, 0.081]), as well as the link between father–child conflict frequency and externalizing problems (*β* = 0.01, 95% CI [0.001, 0.034]). In serial mediation, father–child conflict frequency indirectly affected internalizing problems both via the pathway from maternal psychological control to basic psychological need (*β* = 0.01, 95% CI [0.001, 0.016]) and via the pathway from paternal psychological control to basic psychological need (*β* = 0.01, 95% CI [0.001, 0.019]); these same serial pathways also had significant indirect effects on externalizing problems (*β* = 0.01, 95% CI [0.001, 0.016]; *β* = 0.01, 95% CI [0.001, 0.017], respectively). Mother–child conflict frequency indirectly affected internalizing problems (*β* = 0.02, 95% CI [0.009, 0.039]) and externalizing problems (*β* = 0.02, 95% CI [0.009, 0.031]) through the serial pathway from maternal psychological control to basic psychological needs, and it also negatively predicted life satisfaction through this same pathway (*β* = −0.02, 95% CI [−0.045, −0.011]). In addition, father–child conflict frequency indirectly and negatively predicted life satisfaction (*β* = −0.01, 95% CI [−0.016, −0.001]) and prosocial behavior (*β* = −0.01, 95% CI [−0.021, −0.001]) via the serial pathway from paternal psychological control to basic psychological need.

For secondary school students, T1 conflict frequency did not significantly predict T3 internalizing problems, life satisfaction, or prosocial behavior directly, but T1 father–child conflict frequency did significantly and positively predict T3 externalizing problems. In specific indirect effects, basic psychological needs significantly mediated all associations between mother–child conflict frequency and the four adjustment indicators: internalizing problems (*β* = 0.05, 95% CI [0.002, 0.098]), externalizing problems (*β* = 0.04, 95% CI [0.002, 0.080]), life satisfaction (*β* = −0.03, 95% CI [−0.073, −0.003]), and prosocial behavior (*β* = −0.05, 95% CI [−0.085, −0.013]). Paternal psychological control significantly mediated the link between father–child conflict frequency and prosocial behavior (*β* = 0.03, 95% CI [0.001, 0.065]), and maternal psychological control significantly mediated the link between mother–child conflict frequency and prosocial behavior (*β* = −0.09, 95% CI [−0.142, −0.045]). For serial mediation, father–child conflict frequency indirectly affected internalizing problems via the pathway from paternal psychological control to basic psychological need (*β* = 0.01, 95% CI [0.002, 0.034]) and via the pathway from maternal psychological control to basic psychological need (*β* = 0.02, 95% CI [0.013, 0.046]); the serial pathway from paternal psychological control to basic psychological needs also further affected externalizing problems (*β* = 0.01, 95% CI [0.002, 0.027]) and prosocial behavior (*β* = −0.02, 95% CI [−0.034, −0.002]). Mother–child conflict frequency indirectly affected externalizing problems (*β* = 0.03, 95% CI [0.007, 0.048]), life satisfaction (*β* = −0.02, 95% CI [−0.056, −0.010]), and prosocial behavior (*β* = −0.03, 95% CI [−0.048, −0.014]) through the serial pathway from maternal psychological control to basic psychological need.

## 4. Discussion

This three-wave longitudinal study systematically examined the longitudinal effects of father–child and mother–child conflict frequency on internalizing problems, externalizing problems, life satisfaction, and prosocial behavior among primary and secondary school students and tested the independent and serial mediating roles of parental psychological control and basic psychological need satisfaction. The findings provide new empirical evidence for the multi-layered mechanisms linking parent–child conflict to children and adolescents’ psychological adjustment, while also revealing similarities and differences in these mechanisms across the two school stages.

### 4.1. The Mediating Role of Parental Psychological Control

Parental psychological control emerged as a key independent mediator linking parent–child conflict frequency to students’ psychological adjustment, with pronounced stage-specific and parent-specific patterns, thereby supporting H1 and H4.

Among primary school children, maternal psychological control constituted the more prominent pathway. Mother–child conflict frequency, through maternal psychological control, significantly predicted increases in both internalizing and externalizing problems and tended to suppress prosocial behavior. This finding highlights mothers’ core role in daily parenting of primary school children; mothers typically shoulder more routine care and emotional support, and frequent mother–child conflict may particularly elicit guilt induction, love withdrawal, and other psychologically controlling tactics aimed at restoring behavioral control (Soenens & Vansteenkiste, 2010). The cumulative use of such strategies may directly impair children’s emotion regulation and social motivation, manifesting as elevated internalizing and externalizing problems and dampened prosocial tendencies. Notably, the indirect path from mother–child conflict to life satisfaction via maternal psychological control was not significant, suggesting that primary schoolers’ overall life evaluations may rely on a broader network of supports inside and outside the family, such that a single dimension of mother–child interaction is insufficient to substantially alter their subjective well-being (Suldo & Huebner, 2004).

By contrast, father–child conflict frequency did not significantly affect any adjustment indicator through paternal psychological control itself, yet it significantly predicted increased externalizing problems via maternal psychological control, with similar trends for internalizing problems and reduced prosocial behavior. This pattern is consistent with the spillover hypothesis within family systems theory, wherein tension in the father–child or marital subsystem spills over and erodes the quality of mother–child interactions (Cox & Paley, 1997). Frequent father–child conflict may heighten mothers’ parenting stress amid a tense overall family climate, leading them to rely more heavily on psychological control (Zhou et al., 2025). Hence, paternal negative influences during the primary years may be partially transmitted indirectly through mothers’ parenting.

Upon entering secondary school, mother–child conflict frequency no longer predicted internalizing or externalizing problems through maternal psychological control; instead, it solely predicted declines in prosocial behavior. This shift may indicate that as adolescents’ autonomy strivings intensify, the immediate power of maternal psychological control to provoke internalizing and externalizing symptoms weakens, yet its suppressive effect on prosocial motivation persists. When mothers rely on guilt induction or love withdrawal, secondary schoolers may redirect psychological resources toward resisting control and defending their autonomy, thereby diminishing empathic concern and helping behavior (Mabbe et al., 2018). Concurrently, the mediating role of paternal psychological control became markedly prominent. Father–child conflict frequency, through paternal psychological control, positively predicted declines in prosocial behavior. This finding aligns with You et al. (2025), who reported that fathers’ psychologically controlling strategies—such as devaluation, dismissiveness, and conditional regard—inhibit adolescents’ autonomous motivation and consequently weaken their prosocial engagement. Adolescence is centrally defined by identity construction and surging autonomy needs (Deci & Ryan, 2000). During this period, paternal psychological control may directly threaten the basic need for autonomy, driving adolescents to withdraw or respond passively in prosocial situations due to a lack of intrinsic motivation.

### 4.2. The Mediating Role of Basic Psychological Needs

Basic psychological need served as a significant independent mediator between mother–child conflict and all four adjustment indicators only among secondary school students, partially supporting H2.

Specifically, mother–child conflict frequency predicted elevated internalizing and externalizing problems and diminished life satisfaction and prosocial behavior through reduced need satisfaction. According to self-determination theory (Ryan & Deci, 2000), the satisfaction or frustration of the basic needs for autonomy, competence, and relatedness, which constitute the foundational psychological nutrients, is the direct mechanism translating environmental factors into adjustment outcomes. During the critical transition toward autonomy and independent identity, secondary school students’ need for autonomy is especially sensitive and fragile. Frequent mother–child conflict directly challenges this need while simultaneously conveying implicit disapproval of their social competence (“you are not mature enough to manage yourself”) and eroding the secure emotional bond (La Guardia & Patrick, 2008). The resulting triple need frustration may create a core proximal risk that pervades multiple adjustment outcomes. Moreover, the detrimental effect of mother–child conflict on basic need satisfaction spilled over to emotional experience, subjective well-being, and prosocial tendencies, reflecting the integrative function of basic psychological needs as a core psychological resource (Vansteenkiste & Ryan, 2013). The nonsignificance of this pathway in primary school further indicates that as children enter adolescence, their perceptions of relationship quality may be more directly and deeply converted into actual internal need states, which in turn are translated into a wide range of adjustment outcomes.

### 4.3. The Serial Mediating Role of Parental Psychological Control and Basic Psychological Needs

A central finding of this study is that the hypothesized serial pathway, parent–child conflict frequency → parental psychological control → basic psychological needs → psychological adjustment outcomes, was supported, providing longitudinal evidence for a cascading mechanism proceeding from distal context to proximal parenting behavior, to internal psychological needs, and to adjustment outcomes. This advances understanding of how parent–child conflict shapes child and adolescent development.

#### 4.3.1. Serial Mediation Pathways in Primary School

In the primary school model, both mother–child and father–child conflict frequency significantly predicted internalizing and externalizing problems through the serial pathway of “maternal psychological control → basic psychological need” and also showed a tendency to suppress life satisfaction and prosocial behavior. Additionally, father–child conflict frequency significantly influenced all four adjustment indicators through the serial pathway of “paternal psychological control → basic psychological needs.”

These findings highlight several key features. First, maternal psychological control functioned as a central mediating hub, channeling both the direct effects of mother–child conflict and the spillover effects of father–child conflict. Second, paternal psychological control already operated as an independent serial mediator, indicating that even during the primary school years, paternal parenting influences are far from peripheral as traditionally assumed (Lamb & Lewis, 2013). Third, the robust serial mediation suggests that parent–child conflict frequency may solidify into stable psychological control tendencies in family interactions (L. Sun et al., 2021). Once formed, these psychologically controlling behaviors may continuously erode children’s basic psychological needs, thereby compromising their psychological adjustment.

#### 4.3.2. Serial Mediation Pathways in Secondary School

Compared with primary school, the serial mediation pathways in secondary school yielded richer findings with greater parental role differentiation. As in the primary school years, maternal psychological control continued to act as a key mediator. Through the serial pathway of maternal psychological control leading to basic psychological need satisfaction, mother–child conflict frequency significantly predicted externalizing problems, life satisfaction, and prosocial behavior. Concurrently, father–child conflict frequency also significantly predicted internalizing problems and showed a trending effect on life satisfaction via this same pathway. This pattern indicates that even during adolescence, mothers retain a hub position in the family, such that both mother–child and father–child conflict can impair basic psychological need satisfaction through maternal psychological control and consequently relate to adjustment difficulties (Piotrowska et al., 2016).

Meanwhile, father–child conflict frequency, through the serial pathway of paternal psychological control leading to frustration of basic psychological needs, significantly predicted increases in internalizing and externalizing problems and decreases in prosocial behavior, with marginal effects on life satisfaction. This pattern reflects a developmental shift in paternal influence from limited and indirect during the primary years to pervasive and direct in secondary school.

This shift can be understood from two perspectives. Developmentally, adolescence entails intense motivational dynamics for autonomy, making secondary schoolers highly sensitive to any controlling messages (Deci & Ryan, 2000). In traditional family structures, fathers often embody authority and norms, such that paternal psychological control can deliver a more severe blow to adolescents’ need for autonomy than maternal control (Barber et al., 2012). Structurally, a central issue in adolescent–parent conflict is the renegotiation of autonomy and responsibility, which tends to unfold more intensely and directly within the father–child relationship (Smetana et al., 2006). When father–child conflict prompts greater paternal psychological control, the resulting frustration of basic psychological need may progressively undermine adolescent adjustment over time. Consistent with Levitt et al. (2020), who documented that paternal influence transitions from early indirect and contextual to more direct and confrontational as children enter adolescence, our findings illustrate this developmental transformation.

In sum, the serial mediation models suggest that parent–child conflict frequency is linked to adjustment outcomes primarily through indirect pathways rather than as a sole direct predictor. Specifically, conflict frequency shapes parental psychological control and erodes children’s basic psychological need satisfaction, which in turn predict psychological adjustment. This finding offers empirical grounding for integrating family stress theory (Patterson, 2002) and self-determination theory (Deci & Ryan, 2000), with the former delineating the external stressor rooted in family interaction and the latter revealing the psychological mediating mechanism through which that stress is associated with individual adjustment outcomes.

### 4.4. The Practical Significance of Small Indirect Effects

Notably, all completely standardized indirect effects in this study fell within a small range (absolute values = 0.01 to 0.09). While the large sample size (N > 2000) afforded ample power to detect these effects as statistically significant, their practical significance merits careful consideration. In family and developmental research, standardized indirect effects are often modest in size, as distal stressors must act through chains of intervening mechanisms to influence broad adjustment outcomes (Götz et al., 2022). As Abelson (1985) argued, a small effect can be consequential when it represents a process that is recurrent and cumulative. Parent–child conflict is precisely such a process: it is an ongoing, everyday stressor, so a small effect observed at a single measurement point may accumulate over repeated exposure to produce meaningful long-term impacts (McCartney & Rosenthal, 2000). From a public health perspective, even modest effects on prevalent outcomes such as adolescent internalizing and externalizing problems can translate into substantial population-level benefits, particularly when the mediating mechanisms, psychological control, and basic psychological needs are modifiable through intervention (Götz et al., 2022). Indeed, interventions that reduce psychological control or restore basic need satisfaction could generate cascading improvements that amplify the downstream benefits for youth adjustment. Therefore, the small indirect effects identified here should not be dismissed, as they pinpoint viable and modifiable intervention targets. Future research should search for moderators that strengthen these pathways and should replicate the mediation model in independent samples to further substantiate the robustness and practical relevance of the observed effect sizes (Preacher & Kelley, 2011).

### 4.5. Significance, Practical Implications, and Limitations

By longitudinally differentiating father–child and mother–child conflict, this study deepens our understanding of the multidimensional structure of parent–child conflict, moving beyond the common treatment of conflict as a unitary construct. Further, the serial mediation model grounded in self-determination theory delineates a core transmission mechanism (parental psychological control → basic psychological needs) through which conflict affects adjustment, offering a testable integrative framework for future work. The documented stage-specific differences, particularly the pivotal transformation of paternal psychological control during adolescence, also contribute new longitudinal evidence to developmental family psychology. In practice, the findings carry clear implications for family education and school mental health services. First, interventions for high-conflict families should prioritize identifying and correcting parental psychological control, promoting a shift toward autonomy-supportive parenting. Second, school mental health programs should target basic psychological needs as a core intervention goal, using systematic activities and curricula to meet students’ needs for autonomy, competence, and relatedness and thereby help at-risk students restore damaged psychological resources.

Several limitations must be acknowledged. First, the exclusive use of self-report measures may inflate shared method variance; future studies should incorporate multi-informant designs and dynamic methods to capture fluctuations in conflict and adjustment. Second, caution is warranted when generalizing these findings, given the sample’s limited geographical diversity; future research should recruit larger and more geographically diverse samples to examine the robustness and broader applicability of the observed patterns. Third, Time 1 parent–child conflict data were collected retrospectively at Time 2, risking recall bias and shared method variance with Time 2 measures. Although retrospective reports of short-term, high-frequency concrete behaviors show acceptable validity (Hardt & Rutter, 2004), future fully prospective multi-wave studies are needed to replicate these findings and rule out retrospective confounds.

## 5. Conclusions

This three-wave longitudinal study systematically examined the mechanisms through which father–child and mother–child conflict frequency influence internalizing problems, externalizing problems, life satisfaction, and prosocial behavior among primary and secondary school students. The main conclusions are as follows:

(1) Parental psychological control and basic psychological need served as important independent mediators, with pronounced stage-specific patterns. In primary school, maternal psychological control was the core mediator; in secondary school, basic psychological need emerged as a comprehensive mediator, and the independent mediating role of paternal psychological control was substantially strengthened, reflecting a developmental shift from indirect influence to direct effect.
(2) The serial mediation pathway “parent–child conflict frequency → parental psychological control → basic psychological needs → psychological adjustment” was robustly established across both school stages. This indicates that parent–child conflict, as a distal stressor, contributes to adjustment difficulties only through the successive transmission of maladaptive parenting behaviors and the erosion of psychological resources.

These findings provide longitudinal empirical support for integrating family stress theory and self-determination theory. Moreover, they pinpoint the core targets for family education guidance and school psychological intervention: reducing parental psychological control and restoring children’s basic psychological need satisfaction are the key pathways for helping families recover from conflict and promoting students’ positive adjustment.

## Figures and Tables

**Table 1:** Descriptive statistics and correlation coefficients.

	*M* ± *SD*		*t*	1	2	3	4	5	6	7	8	9
	Primary School	Secondary School										
1. T1FFCC	2.39 ± 1.04	2.28 ± 1.02	2.72 **	1	0.84 ***	0.35 ***	0.32 ***	−0.19 ***	0.17 ***	0.16 ***	−0.13 ***	−0.08 *
2. T1FMCC	2.34 ± 1.07	2.27 ± 1.05	1.45	0.87 ***	1	0.32 ***	0.39 ***	−0.24 ***	0.18 ***	0.18 ***	−0.13 ***	−0.13 ***
3. T2PPC	2.53 ± 1.02	2.57 ± 1.03	−0.96	0.41 ***	0.40 ***	1	0.76 ***	−0.31 ***	0.21 ***	0.16 ***	−0.16 ***	−0.08 *
4. T2MPC	2.48 ± 1.04	2.47 ± 1.02	0.34	0.39 ***	0.42 ***	0.82 ***	1	−0.35 ***	0.26 ***	0.22 ***	−0.20 ***	−0.11 **
5. T2BPN	3.31 ± 0.53	3.24 ± 0.56	2.86 **	−0.28 ***	−0.29 ***	−0.34 ***	−0.36 ***	1	−0.41 ***	−0.32 ***	0.32 ***	0.15 ***
6. T3IP	1.56 ± 0.38	1.67 ± 0.41	−6.64 ***	0.21 ***	0.24 ***	0.26 ***	0.28 ***	−0.34 ***	1	0.52 ***	−0.42 ***	−0.09 **
7. T3EP	1.61 ± 0.36	1.67 ± 0.34	−4.25 ***	0.26 ***	0.28 ***	0.29 ***	0.31 ***	−0.36 ***	0.78 ***	1	−0.38 ***	−0.13 ***
8. T3PB	2.40 ± 0.46	2.34 ± 0.45	2.51 ***	−0.13 ***	−0.12 ***	−0.14 ***	−0.17 ***	0.29 ***	−0.31 ***	−0.45 ***	1	0.28 ***
9. T3LS	4.71 ± 1.12	4.29 ± 1.03	9.41 ***	−0.08 *	−0.07 ***	−0.09 **	−0.11 ***	0.25 ***	−0.13 ***	−0.23 ***	0.36 ***	1

Note. Correlations below the diagonal represent primary school dyads; above the diagonal represent secondary school dyads. T1 = Time 1, T2 = Time 2, T3 = Time 3. FFCC = frequency of father–child conflicts, FMCC = frequency of mother–child conflicts, PPC = paternal psychological control, MPC = maternal psychological control, BPN = basic psychological need, IP = internalizing problems, EP = externalizing problems, PB = prosocial behavior, LS = life satisfaction. *** *p* < 0.001, ** *p* < 0.01, * *p* < 0.05. All subsequent tables follow this convention.

**Table 2:** Results of measurement invariance testing.

	Model	χ^2^ (*df*)	CFI	TLI	RMSEA	Comparison	∆CFI	∆RMSEA
FFCC	M1	419.13 (54)	0.972	0.971	0.079			
	M2	436.24 (55)	0.971	0.971	0.080	M2–M1	−0.001	0.001
	M3	480.11 (63)	0.968	0.972	0.078	M3–M2	−0.003	−0.002
FMCC	M1	419.55 (52)	0.976	0.974	0.080			
	M2	439.67 (53)	0.974	0.973	0.082	M2–M1	−0.002	0.002
	M3	472.31 (61)	0.973	0.975	0.079	M3–M2	−0.001	−0.003
PPC	M1	65.62 (10)	0.994	0.988	0.071			
	M2	66.70 (11)	0.994	0.988	0.070	M2–M1	0.000	−0.001
	M3	76.06 (13)	0.993	0.989	0.067	M3–M2	−0.001	−0.003
MPC	M1	103.93 (14)	0.991	0.988	0.077			
	M2	108.50 (15)	0.991	0.987	0.076	M2–M1	0.000	−0.001
	M3	138.40 (19)	0.989	0.988	0.076	M3–M2	−0.002	0.000
BPN	M1	414.03 (60)	0.946	0.936	0.074			
	M2	451.83 (62)	0.941	0.931	0.076	M2–M1	−0.005	0.002
	M3	474.83 (71)	0.939	0.938	0.072	M3–M2	−0.002	−0.004
IP	M1	536.60 (76)	0.960	0.953	0.075			
	M2	572.07 (78)	0.957	0.951	0.076	M2–M1	−0.003	0.001
	M3	633.33 (88)	0.953	0.952	0.075	M3–M2	−0.004	−0.001
EP	M1	609.15 (76)	0.941	0.930	0.080			
	M2	650.26 (78)	0.936	0.926	0.082	M2–M1	−0.005	0.002
	M3	705.24 (88)	0.931	0.930	0.080	M3–M2	−0.005	−0.002
PB	M1	48.12 (10)	0.993	0.985	0.059			
	M2	71.59 (12)	0.988	0.981	0.067	M2–M1	−0.005	0.008
	M3	95.07 (17)	0.985	0.982	0.065	M3–M2	−0.003	−0.002
LS	M1	49.91 (10)	0.989	0.987	0.052			
	M2	69.77 (14)	0.985	0.986	0.058	M2–M1	−0.004	0.006
	M3	95.18 (18)	0.979	0.953	0.063	M3–M2	−0.006	0.005

Note. M1 = configural invariance; M2 = metric invariance; M3 = scalar invariance. FFCC = frequency of father–child conflicts, FMCC = frequency of mother–child conflicts, PPC = paternal psychological control, MPC = maternal psychological control, BPN = basic psychological needs, IP = internalizing problems, EP = externalizing problems, PB = prosocial behavior, LS = life satisfaction.

**Table 3:** Indirect pathways of parental–child conflict frequency on students’ psychological adjustment (significant pathways).

Educational Stage	Paths	*β*	95% CI
primary school	FMCC → MPC → IP	0.04	[0.010, 0.088]
	FFCC → MPC → EP	0.01	[0.001, 0.034]
	FMCC → MPC → EP	0.03	[0.007, 0.081]
	FFCC → MPC → BPN → IP	0.01	[0.001, 0.016]
	FFCC → PPC → BPN → IP	0.01	[0.001, 0.019]
	FMCC → MPC → BPN → IP	0.02	[0.009, 0.039]
	FMCC → MPC → BPN → EP	0.02	[0.009, 0.031]
	FFCC → PPC → BPN → EP	0.01	[0.001, 0.017]
	FFCC → MPC → BPN → EP	0.01	[0.001, 0.016]
	FFCC → PPC → BPN → LS	−0.01	[−0.016, −0.001]
	FMCC → MPC → BPN → LS	−0.02	[−0.045, −0.011]
	FFCC → PPC → BPN → PB	−0.01	[−0.021, −0.001]
	FMCC → MPC → BPN → PB	−0.02	[−0.043, −0.001]
secondary school	FMCC → BPN → IP	0.05	[0.002, 0.098]
	FMCC → BPN → EP	0.04	[0.002, 0.080]
	FMCC → BPN → PB	−0.05	[−0.085, −0.013]
	FMCC → BPN → LS	−0.03	[−0.073, −0.003]
	FFCC → PPC → PB	0.03	[0.001, 0.065]
	FMCC → MPC → PB	−0.09	[−0.142, −0.045]
	FFCC → PPC → BPN → IP	0.01	[0.002, 0.034]
	FFCC → MPC → BPN → IP	0.02	[0.013, 0.046]
	FFCC → PPC → BPN → EP	0.01	[0.002, 0.027]
	FMCC → MPC → BPN → EP	0.03	[0.007, 0.048]
	FMCC → MPC → BPN → LS	−0.02	[−0.056, −0.010]
	FFCC → PPC → BPN → PB	−0.02	[−0.034, −0.002]
	FMCC → MPC → BPN → PB	−0.03	[−0.048, −0.014]

Note. FFCC = frequency of father–child conflicts, FMCC = frequency of mother–child conflicts, PPC = paternal psychological control, MPC = maternal psychological control, BPN = basic psychological needs, IP = internalizing problems, EP = externalizing problems, PB = prosocial behavior, LS = life satisfaction.

## Data Availability

The data presented in this study are available on request from the corresponding author due to ethical restrictions (the need to protect participant confidentiality and comply with institutional review board policies).
